# Vertebral osteomyelitis caused by *Chaetomium Globosum* in a young adult: A case report and literature review

**DOI:** 10.1097/MD.0000000000049055

**Published:** 2026-05-29

**Authors:** Ruliang Liang, Ming Peng, Jianying Huang, Weiqun Wang, Ziwen Fang, Guixian Bao, Yaoxin Chen, Rongqiang Yang, Zehong Zhou, Liting Liu, Danqian Lu

**Affiliations:** a Department of Laboratory Medicine, Zhongshan Hospital of Traditional Chinese Medicine Affiliated to Guangzhou University of Chinese Medicine, Zhongshan, Guangdong, People’s Republic of China; b Department of Laboratory Medicine, Zhongshan Hospital of Traditional Chinese Medicine, Zhongshan, Guangdong, People’s Republic of China.

**Keywords:** chaetomium, mycoses, opportunistic Infections, spondylitis

## Abstract

**Rationale:**

*Chaetomium globosum (C. globosum*) is a saprophytic fungus widely distributed in nature, characterized by low clinical pathogenicity but capable of causing severe infections, particularly in immunocompromised hosts or under conducive environmental conditions. Spinal infections attributable to C. globosum are exceedingly rare and often present diagnostic dilemmas due to atypical presentations. This case underscores the importance of considering fungal etiologies in spinal pathologies. Furthermore, the strain mentioned in this study has been appropriately preserved and is available for further research. Researchers interested in investigating the mechanisms underlying its enhanced invasiveness compared to other strains are encouraged to contact the corresponding author.

**Patient concerns:**

A 24-year-old male was admitted with a chief complaint of persistent low back pain lasting for 2 weeks, without significant relief from initial conservative measures, without immune-related diseases or other comorbidities.

**Diagnoses:**

Routine fluid analyses showed no indicators of infection; however, magnetic resonance imaging (MRI) demonstrated cystic lesions localized to the L3 vertebra, raising suspicion for a spinal infectious process. Definitive diagnosis was achieved through tissue culture, which identified C. globosum as the causative pathogen.

**Interventions:**

Initial management employed a dual-therapy approach combining agents aimed at enhancing circulation with conventional analgesics and antiinflammatory drugs. Upon microbiological confirmation, targeted antifungal therapy with itraconazole was initiated.

**Outcomes:**

The patient exhibited progressive clinical improvement, marked by substantial pain reduction during hospitalization, and attained stable recovery with resolution of symptoms by the time of discharge.

**Lessons:**

This case highlights a rare spinal C. globosum infection in an immunocompetent adult and underscores the importance of considering fungal pathogens in the differential diagnosis of unexplained spinal lesions. Early tissue biopsy with histopathological and molecular analysis is essential for definitive diagnosis and guiding appropriate antifungal therapy.

## 1. Introduction

Spinal infections represent a severe pathology characterized by low back pain, restricted mobility, and neurological deficits, frequently accompanied by systemic symptoms such as fever and fatigue. Imaging reveals vertebral bony changes and paravertebral soft tissue swelling. Diagnosis relies on comprehensive assessment of clinical manifestations, radiological findings, and laboratory tests.^[1,2]^ Spinal infections typically arise from bacterial, fungal, or other pathogens and may cause severe complications including spinal cord injuries and permanent disability.^[3,4]^ Bacterial etiologies predominate, particularly Staphylococcus aureus infections, which are more frequent in patients over 70 years old^[5,6]^ Fungal spinal infections, though less common, primarily affect immunocompromised individuals due to conditions like diabetes, corticosteroid use, chemotherapy, or malnutrition,^[7]^ or may develop secondary to disseminated fungal infections.^[8]^ Spinal fungal infections present significant therapeutic challenges and often result in terrible outcomes when established.^[9]^ Current management includes surgical debridement and atifungal drugs likes azole or amphotericin B,^[10]^ with early diagnosis being critical for prognosis.^[11]^

This case documents a spinal fungal infection caused by Chaetomium globosum. Chaetomium globosum, a saprophytic ascomycete, exists as mold in soil and produces infectious spores under favorable conditions. Human infections primarily follow spore inhalation, causing pulmonary or disseminated disease. Reported cases involve nails,^[12,13]^ skin,^[14,15]^ cornea,^[16]^ lungs,^[17]^ and in immunocompromised hosts—encephalitis^[18]^ or systemic infection.^[19]^ Notably, spinal involvement by C. globosum has never been reported in immunocompetent young adults.

As the first documented case of spinal C. globosum infection in a non-immunocompromised youth, this report provides novel insights into invasive fungal management in immunologically intact populations and underscores the importance of evaluating environmental exposures in young adults.

## 2. Cases presentation

**Figure 1.**
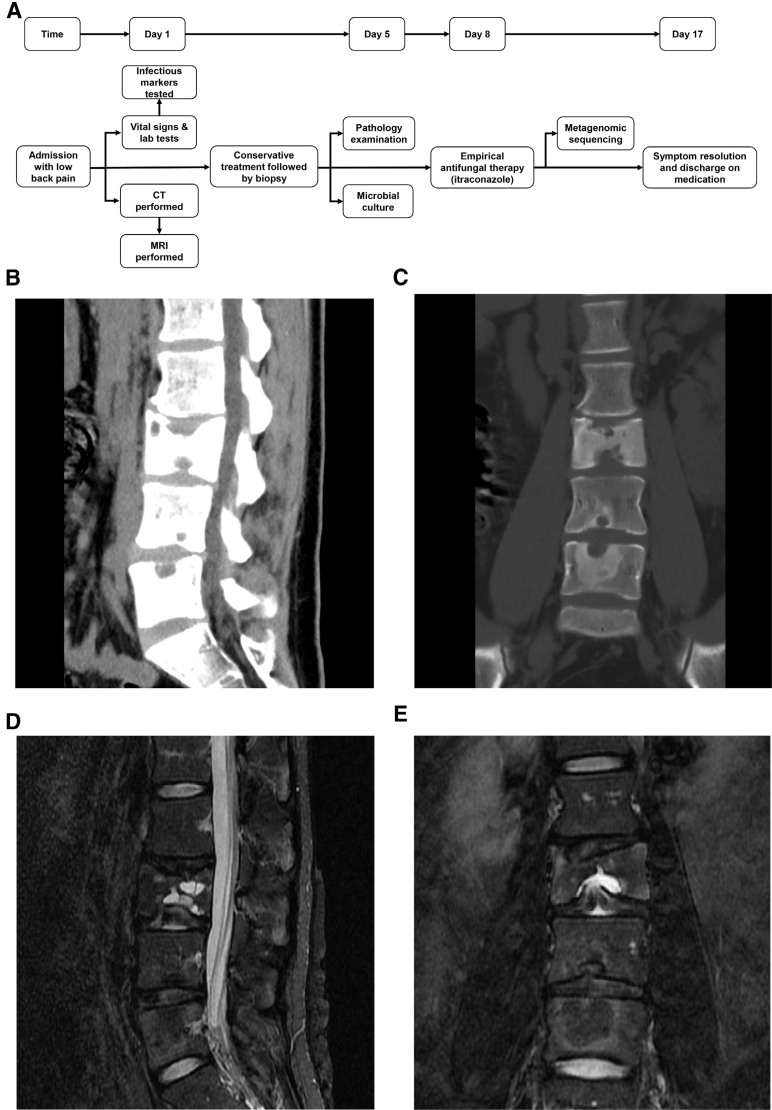
CT and MRI results. (A) Patient admission flow chart. (B, C) Sagittal (A) and coronal (B) CT images of the lumbar spine demonstrate: Multiple Schmorl nodes in the L3-L5 vertebral bodies. Disc bulging at L3/4 and L4/5 levels Mild disc protrusion at L5/S1. (D, E) Sagittal (C) and coronal (D) MRI of the lumbar spine reveal: Osteolytic destruction in L3 and L5 vertebrae, suggestive of infection. Depression of the inferior endplate at L4 with surrounding bone marrow edema, consistent with an acute Schmorl node. Disc degeneration at L2/3, L3/4, and L4/5 levels. Disc bulging at L3/4 and L4/5.

This case report, representing the first reported instance of Chaetomium osteomyelitis of the spine, was conducted at Zhongshan Hospital of Traditional Chinese Medicine Affiliated to Guangzhou University of Chinese Medicine, Zhongshan, China from February 18, 2023, to March 7, 2023 (Fig. 1A). A 24-year-old male graduate student of Asian descent presented with a 2-week history of low back pain. The pain was insidious in onset, progressively worsening, and was described as a persistent, dull ache localized to the lumbar region without radiation to the lower limbs. It was consistently aggravated by physical activity and alleviated with rest. Previous analgesic treatment (unspecified) was ineffective. On admission, he was conscious but lethargic, reporting localized back pain without intermittent claudication, sensory disturbances, fever, cough, chest/abdominal pain, or abnormal sweating. Vital signs included pulse 80 bpm, respirations 20/min, and BP 111/70 mm Hg. Spinal inspection showed loss of lumbar lordosis, paraspinal muscle tenderness at L3/4 and L4/5, and normal lower extremity motor/sensory function without pathological reflexes. Special tests, including the straight leg raise test (70° bilaterally, negative), and tests for pathological reflexes (Babinski, clonus) were negative. Routine fluid analysis revealed unremarkable hepatic/renal function and infection biomarkers, with normal white blood cell counts and erythrocyte sedimentation rate (Table 1). These normal neurological findings, along with the absence of systemic symptoms, helped exclude common etiologies such as acute disc herniation with significant nerve root compression, spinal stenosis, or a systemic inflammatory arthropathy at the initial presentation.

**Table 1:** Routine fluid analysis in clinical laboratory.

Biochemical parameters	Result
ALT U/L	26
AST U/L	22
TP g/L	67.7
Alb g/L	43.8
BUN mmol/L	3.06
Cr umol/L	72
TCO_2_ mmol/L	27.6
Hematological Parameters	
WBC 109/L	4.54
Neu# 109/L	2.66
Lym# 109/L	1.27
Mon# 109/L	0.38
Eos# 109/L	0.15
Bas# 109/L	0.08
RBC 1012/L	4.58
PLT 109/L	307
ESR mm/H	8
Infection Biomarker	
PCT ng/mL	0.03
CRP mg/L	1

Bas# = Basophil Count, CRP# = C-Reactive Protein, Eos# = Eosinophil Count, ESR = Erythrocyte Sedimentation Rate, Lym# = Lymphocyte Count, Mon# = Monocyte Count, Neu# = Neutrophil Count, PCT = Procalcitonin, PLT = Platele, RBC = Red Blood Cell, WBC = White Blood Cell.

Imaging revealed L2/3 disc degeneration with a Schmorl node at L3, mild abnormal density foci in L3-L5 vertebrae with L5/S1 central herniation (Computed Tomography (CT); Fig. 1B-C), and lytic bone destruction with altered signal intensity was demonstrated in the L3 and L5 vertebrae, suggestive of infection. L4 endplate depression/marrow edema suggesting Schmorl node, plus disc degeneration/bulging at L2/3-L4/5 (MRI; Fig. 1D-E), collectively indicating inflammatory/infectious pathology. Infectious spondylodiscitis characteristically manifests as contiguous involvement of 2 adjacent vertebral bodies and the intervening intervertebral disc. In contrast, uncomplicated degenerative disc disease typically does not present with extensive vertebral bone marrow edema or enhancement. Furthermore, although osseous metastases may appear as lytic, sclerotic, or mixed destructive lesions, they classically spare the intervertebral disc. The pattern of bone marrow involvement in metastatic disease is often focal or skip, rather than the contiguous spread observed in infection. Therefore, the imaging findings in this case are more suggestive of infectious spondylodiscitis. Initial management included aceclofenac (0.1 g bid), ranitidine (0.15 g bid), bed rest.

**Figure 2.**
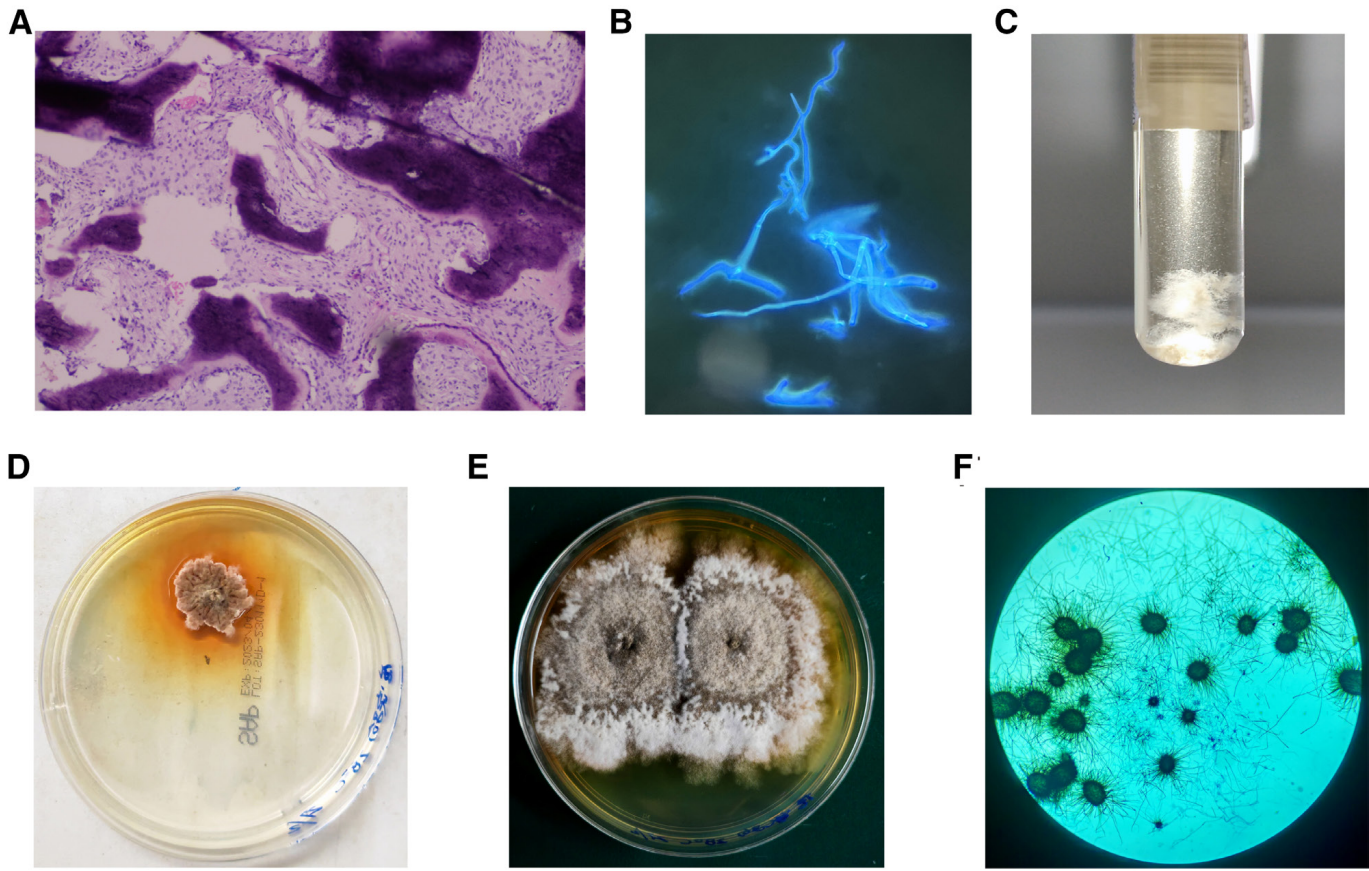
Histopathological and bacteriological result. (A) Histopathology (H&E staining): Fragmented bone trabeculae with scattered lymphocytes infiltration. (B) Fungal immunofluo-rescence staining of tissue smear: Septate hyphae detected. (C) Bacterial culture (nutrient broth, 3 days): White microbial colonies observed. (D-E) Chaetomium globosum colonies: Sabouraud dextrose agar (SDA), 28°C, 5 days (D) and 9 days (E). (F) Microscopy of Chaetomium globosum: Lactophenol cotton blue staining (× 100 magnification).

After admission on day 5, pathological examination and bacterial culture were conducted on bone and fibrous tissue samples obtained via lumbar puncture, and conservative therapy was discontinued due to symptomatic relief. Biopsy showed reduced bone/fibrous tissue with lymphocytic infiltration (Fig. 2A). Gram staining was negative, but fluorescent staining revealed fungal hyphae (Fig. 2B). Broth culture yielded fungal masses in 3 d (Fig. 2C), and SDA fungal culture revealed brown woolly colonies (Fig. 2D). After 2 weeks of isolation culture, white colonies with subtly visible gray-to-black ascomata specks centrally interspersed were observed, exhibiting a tan to reddish-brown reverse (Fig. 2E). Microscopy identified septate hyphae and globose ascomata with brown setae containing evanescent-walled ascospores (Fig. 2F). Metagenomic sequencing confirmed C. *globosum* (Supplemental material 1). Further history noted this patient residence in a humid, mold-infested basement dormitory with minimal sunlight exposure, sedentary habits, hyperhidrosis, and recurrent skin eruptions.

Final diagnosis was lumbar fungal osteomyelitis. Itraconazole (100 mg daily for 4 months) was initiated. By 2 weeks, mobility improved permitting discharge. The patient reported that Subsequent imaging (CT/MRI) revealed no lumbar spine disease progression. During recent follow-up, the patient reported residual mild intermittent low back discomfort.

## 3. Review of the literature

We conducted a retrospective analysis of fungal spondylitis cases over the past 2 decades. A systematic literature search was conducted in PubMed using the search query: (“spinal infection” OR “fungal spondylodiscitis” OR “vertebral osteomyelitis”) AND (“fungal” OR “Candida” OR “Aspergillus” OR “Scedosporium”). The search was limited to publications from the past 2 decades. Two independent reviewers screened the full text of all retrieved articles. Studies were excluded if they were case reports lacking: 1) definitive diagnostic evidence for fungal spondylitis (e.g., etiologic culture, histopathology, or reliable molecular detection); 2) a clear description of the treatment regimen; or 3) well-defined outcome measures with follow-up duration. Following this screening, data from 96 patients were included for statistical evaluation, comprising 85 survivors (88.5%) and 11 deceased patients (11.5%). Baseline characteristics—including demographics, medical history, and treatment factors—were compared between groups to assess prognostic associations (Table 2; Supplemental material 2).

**Table 2:** Baseline characteristics of the reviewed literature.

Characteristics	Alive	Dead	*P* value	Statistic	Method
n	85	11			
Age, median (IQR)	56 (44, 65)	53 (42.5, 71)	.809		Wilcoxon
Gender, n (%)			.837	0.042	Yates’ correction
M	61 (63.5%)	7 (7.3%)			
F	24 (25%)	4 (4.2%)			
History of immunosuppression/chronic diseases, n (%)			.794	1.026	Yates’ correction
NO	47 (49%)	7 (7.3%)			
YES	32 (33.3%)	4 (4.2%)			
No mention	6 (6.2%)	0 (0%)			
Recent major surgery/ICU hospitalization/infection-related medication, n (%)			.798	0.449	Yates’ correction
YES	71 (74%)	10 (10.4%)			
NO	13 (13.5%)	1 (1%)			
No mention	1 (1%)	0 (0%)			
Infection site, n (%)			.048	6.086	Yates’ correction
Lumbar	49 (51%)	3 (3.1%)			
Thoracic	23 (24%)	7 (7.3%)			
Cervical	13 (13.5%)	1 (1%)			
Surgical, n (%)			.224	1.478	Yates’ correction
NO	17 (17.7%)	0 (0%)			
YES	68 (70.8%)	11 (11.5%)			
Antifungal medication, n (%)			.393	7.356	Yates’ correction
Amphotericin B	8 (8.3%)	2 (2.1%)			
Voriconazole	27 (28.1%)	3 (3.1%)			
Fluconazole	21 (21.9%)	0 (0%)			
Combination Therapy	21 (21.9%)	4 (4.2%)			
Itraconazole	5 (5.2%)	1 (1%)			
Micafungin	1 (1%)	1 (1%)			
Caspofungin	1 (1%)	0 (0%)			
Andamycin	1 (1%)	0 (0%)			

IQR = interquartile range.

**Figure 3.**
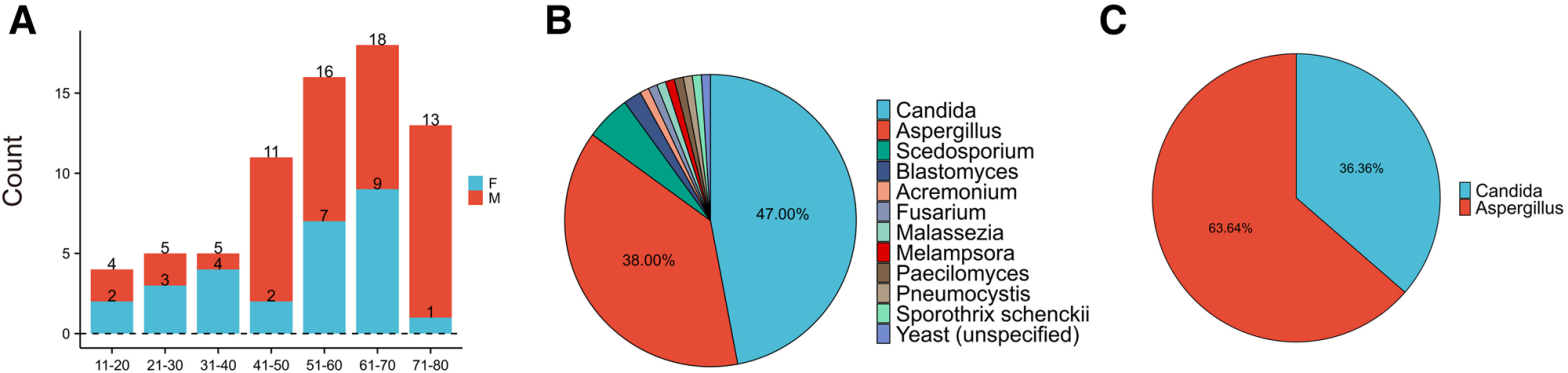
Age and pathogen distribution in the literature review. (A) Histogram showing the distribution of cases by gender and age group. (B) Pie chart illustrating the overall distribution of fungal species. (C) Pie chart displaying the distribution of fungal species among non-survivors.

No significant age difference emerged between survivors (median 56 years, IQR 44–65) and dead (median 53 years, IQR 42.5–71; *P* = .809), indicating age was not a pivotal prognostic factor. Gender distribution also showed no intergroup disparity (*P* = .837): males constituted 63.5% (61/85) and females 25% (24/85) in survivors, vs 7.3% (7/11) males and 4.2% (4/11) females in dead (χ^2^ = 0.042). Visualization of age and gender distribution (Fig. 3A) revealed male predominance (72%) over females (28%), with a male-to-female ratio of 2.57:1. This disparity intensified in older cohorts: the 71–80 age group showed 13 males vs 1 female (ratio 13:1). This aligns with 1 of the points discussed in our reviewed literature regarding potential host factors: differences in host susceptibility may be associated with gender. It has been suggested that male patients might be more likely to have Burkholderia cepacia complex isolated, an infection that is also associated with an immunocompromised state.^[20]^ However, given the lack of a statistically significant difference in gender distribution between the survivor and deceased groups, this descriptive observation cannot be interpreted as evidence for increased susceptibility to or poorer prognosis of fungal spondylitis in males. Further investigation with larger sample sizes is warranted to validate this trend.

Age distribution analysis indicated spinal fungal infections primarily affect middle-aged and elderly populations, with 77% of cases occurring in patients over 41 years. The 51–70 age range represented the highest incidence group. Conversely, only 23 cases involved patients under 40, with relatively balanced gender distribution in younger subgroups, implying that special exposure factors may outweigh baseline immune status in this demographic. Our case, a young patient without immunosuppression, further underscores the potential significance of environmental exposures in younger individuals.

This age pattern aligns with established epidemiological profiles of spinal fungal infections. Literature indicates these infections correlate strongly with immune status, frequently occurring in immunocompromised hosts.^[21]^ Middle-aged and older males often present with comorbidities like diabetes,^[22]^ a recognized risk factor that impairs immune function and elevates fungal infection susceptibility. Age-related immunosenescence may also contribute to higher incidence in older adults.

No significant prognostic differences emerged regarding immunosuppression/chronic disease history (*P* = .795), recent major surgery/ICU admission/antibiotic exposure (*P* = .799), or surgical intervention (*P* = .224). Infection site was the sole variable associated with outcome (*P* = .048). Lumbar involvement predominated in survivors (51%, 49/85), whereas thoracic infections were more frequent in dead (7.3%, 7/11 vs 3.1% lumbar infections). Thoracic involvement may confer higher mortality due to anatomical complexity or treatment challenges (χ^2^ = 6.086). However, due to limitations in the sample size and the granularity of data available from the included literature, a multivariate analysis to adjust for other potential confounding factors was not feasible. Consequently, this finding should be interpreted as a preliminary observation and a potential prognostic clue that requires validation through multivariate analysis in future studies with larger cohorts, rather than as an independent determinant of outcome.

Antifungal regimens showed no mortality association (*P* = .393). Voriconazole (28.1%) and fluconazole (21.9%) were most used in survivors, while combination therapy was more common in dead (4.2%). The absence of fluconazole in dead may suggest either its protective effect or selective use in less severe cases.

Pathogen distribution analysis (Fig. 3B) identified Candida (47 cases) and Aspergillus (38 cases) as predominant pathogens, consistent with typical invasive fungal infection patterns in immunocompromised hosts.^[23]^ Rare species like Scedosporium and Blastomyces were infrequent. Among dead, Aspergillus outnumbered Candida (Fig. 3C), possibly reflecting its heightened invasiveness and poorer prognosis, as aspergillosis in immunocompromised patients often yields higher mortality due to treatment difficulties and frequent co-infections.^[24]^ Candida infections, while common, may respond better to standard antifungals.^[25]^

## 4. Discussion

This case describes a young, immunocompetent male with low back pain whose diagnosis of spinal infection was challenging due to the absence of classic features such as trauma, systemic symptoms, elevated inflammatory markers, and initially nonspecific imaging findings including degenerative changes and Schmorl nodes. Vertebral osteomyelitis/discitis is relatively uncommon in this demographic; statistical analysis indicates only 9% of reported cases occur in patients aged ≤ 24 years, predominantly caused by Candida or Aspergillus with no prior reports of C. globosum in this age group. This underscores the critical need to include infectious etiologies in the differential diagnosis of back pain in young adults, even when more common conditions like musculoskeletal strain are suspected, as prompt diagnosis is paramount for prognosis.

Etiologically diverse spinal infections include bacterial, tubercular, and fungal pathogens.^[26]^ The patient clinical presentation coupled with L3/L5 vertebral lesions on MRI/CT suggested infectious pathology. Diagnostic models can help differentiate infection types,^[27]^ whereas soft tissue injuries typically manifest localized tenderness without systemic symptoms. But differential diagnosis from infectious osteomyelitis was challenging due to the absence of classic signs like fever, negative blood cultures, and normal inflammatory markers, factors that could lead to misdiagnosis as mechanical back pain or neoplastic processes. This discrepancy between imaging findings and unremarkable lab results prompted consideration of atypical pathogens such as fungi, which often cause minimal systemic inflammation. Correlating radiographic suspicion with histopathological and metagenomic analysis of biopsy specimens was crucial for accurate diagnosis, especially in cases with poor response to empirical antibacterial therapy.

Timely intervention through antibiotic therapy or surgery reduces complication risks.^[28]^ This case achieving significant pain relief prior to definitive diagnosis through antiinflammatory analgesics, symptomatic management, and surgical contingency planning. This multimodal approach simultaneously targets the underlying pathology while alleviating symptoms and improving quality of life. For C. *globosum* infection, antifungals like fluconazole^[17,29]^ and itraconazole^[14,30]^ constitute first-line therapy. Conservative management stabilized the patient condition, permitting discharge with maintenance therapy. Subsequent assessments confirmed no disease progression, though long-term monitoring remains essential due to irreversible vertebral damage.

## 5. Conclusions

This report highlights a rare case of spinal C. globosum infection in an immunocompetent adult, emphasizing the need to consider fungal pathogens in the differential diagnosis of unexplained spinal lesions. Definitive diagnosis relies on early tissue biopsy with histopathological and molecular analysis to guide appropriate antifungal therapy. Environmental mold exposure may be a potential risk factor, but its specific role requires further prospective investigation.

## Acknowledgments

Not applicable.

## Author contributions

**Data curation:** Ruliang Liang, Ming Peng, Jianying Huang, Weiqun Wang, Ziwen Fang, Guixian Bao, Rongqiang Yang, Zehong Zhou, Liting Liu.

**Formal analysis:** Ruliang Liang, Ming Peng.

**Writing – original draft:** Ruliang Liang.

**Visualization:** Yaoxin Chen.

**Conceptualization:** Danqian Lu.

**Writing – review & editing:** Danqian Lu.




